# Macrophage and T-Cell Gene Expression in a Model of Early Infection with the Protozoan *Leishmania chagasi*

**DOI:** 10.1371/journal.pntd.0000252

**Published:** 2008-06-25

**Authors:** Nicholas A. Ettinger, Mary E. Wilson

**Affiliations:** 1 Interdisciplinary Graduate Program in Molecular and Cellular Biology, University of Iowa, Iowa City, Iowa, United States of America; 2 Medical Scientist Training Program, University of Iowa Carver College of Medicine, Iowa City, Iowa, United States of America; 3 Departments of Internal Medicine and Microbiology, University of Iowa Carver College of Medicine, Iowa City, Iowa, United States of America; 4 Department of Epidemiology, University of Iowa College of Public Health, Iowa City, Iowa, United States of America; 5 VA Medical Center, Iowa City, Iowa, United States of America; McGill University, Canada

## Abstract

Visceral leishmaniasis is a potentially fatal infectious disease caused by the protozoan parasite *Leishmania infantum/chagasi* in the New World, or by *L. donovani* or *L. infantum/chagasi* in the Old World. Infection leads to a variety of outcomes ranging from asymptomatic infection to active disease, characterized by fevers, cachexia, hepatosplenomegaly and suppressed immune responses. We reasoned that events occurring during the initial few hours when the parasite encounters cells of the innate and adaptive immune systems are likely to influence the eventual immune response that develops. Therefore, we performed gene expression analysis using Affymetrix U133Plus2 microarray chips to investigate a model of early infection with human monocyte-derived macrophages (MDMs) challenged with wild-type *L. chagasi* parasites, with or without subsequent co-culture with Leishmania-naïve, autologous T-cells. Microarray data generated from total RNA were analyzed with software from the Bioconductor Project and functional clustering and pathway analysis were performed with DAVID and Gene Set Enrichment Analysis (GSEA), respectively. Many transcripts were down-regulated by infection in cultures containing macrophages alone, and the pattern indicated a lack of a classically activated phenotype. By contrast, the addition of autologous Leishmania-naïve T cells to infected macrophages resulted in a pattern of gene expression including many markers of type 1 immune cytokine activation (IFN-γ, IL-6, IL-1α, IL-1β). There was simultaneous up-regulation of a few markers of immune modulation (IL-10 cytokine accumulation; TGF-β Signaling Pathway). We suggest that the initial encounter between *L. chagasi* and cells of the innate and adaptive immune system stimulates primarily type 1 immune cytokine responses, despite a lack of classical macrophage activation. This local microenvironment at the site of parasite inoculation may determine the initial course of immune T-cell development.

## Author Summary

Visceral leishmaniasis (VL) is a potentially fatal vector-borne infectious disease that leads to a variety of outcomes ranging from asymptomatic infection to symptomatic disease. In northeast Brazil, the etiological agent of VL is the protozoan *Leishmania chagasi/infantum*. Active VL is characterized by fevers, weight loss, hepatosplenomegaly and eventually immune suppression. Without treatment, most symptomatic patients die from secondary bacterial or viral super-infection. We hypothesized that a unique immune response to *L. chagasi* is initiated early during the initial interactions between the immune system cells that first encounter the parasite. These include macrophages and T-cells, elements of the innate and adaptive immune systems, respectively. We studied an *in vitro* model of these interactions in which human monocyte-derived macrophages were challenged with *L. chagasi*, and subsequently cultured with Leishmania-naïve, autologous T cells. Using microarray chips, we examined changes in global gene expression induced by these early interactions. Infection did not elicit a classical inflammatory program in macrophages. However, co-culture of infected macrophages and autologous T cells exhibited a pattern of gene expression, including many markers of acute inflammation or a type 1 immune response. These data suggest that early changes at the site of parasite infection would be conducive to the development of a protective type 1 response, followed by modulation of this same response.

## Introduction

Visceral leishmaniasis (VL) is a potentially fatal infectious disease caused by the protozoan parasites *Leishmania chagasi/infantum* in the New or in parts of the Old World, or by *L. donovani* in other regions of the Old World.[1] Infection leads to a variety of outcomes ranging from asymptomatic infection to active disease, which is characterized by fevers, cachexia, hepatosplenomegaly and suppressed immune responses. Without treatment, most symptomatic patients die.[2] Investigations into the mechanism underlying the immunosuppression during acute VL have demonstrated defective antigen-specific proliferation and IFN-γ responses to parasite antigen,[3]–[5] high expression of IL-10 in the spleen and serum of symptomatic VL patients[6]–[10] and high serum levels of IL-4, TGF-β and IL-2 receptor.[11]–[13] *In vitro* infection with Leishmania parasites suppresses macrophage microbicidal responses and IFN-γ pathway signaling,[14]–[17] suggesting that these suppressive changes begin at the earliest stages of infection. Whether this defect in macrophage responses to Leishmania infection is communicated to local adaptive immune cells is not known.

We reasoned that events occurring during the initial few hours when the parasite encounters cells of the innate and adaptive immune systems are likely to influence the eventual immune response that develops. We hypothesized that the parasite would cause unique changes in gene expression in both innate and adaptive cells of the immune system encountered early in infection. To test this hypothesis, we analyzed gene expression with an *in vitro* model using human monocyte-derived macrophages (MDMs) challenged with *L. chagasi* promastigotes with or without subsequent co-culture with Leishmania-naïve, autologous T-cells. Gene expression analysis of RNA harvested from both MDMs alone and the MDM-T cell co-cultures indicated a surprising type 1 inflammatory cytokine response during the earliest stages of parasite invasion into the host.

## Materials and Methods

### Parasites

A Brazilian isolate of *L. chagasi* (MHOM/BR/00/1669) was maintained in hamsters by serial intracardiac injection of amastigotes. Parasites were grown as promastigotes at 26°C in liquid hemoflagellate-modified minimal essential medium and used within 3 weeks of isolation.[18] Parasite sub-cultures were used on day 7 of growth for infections.

### Infection protocol

On day zero, venous blood was drawn from four healthy, US resident adult male volunteers ages 24–64 in accordance with the human subjects guidelines approved by the University of Iowa Institutional Review Board. None of the donors have been exposed to *Leishmania*. Written consent was obtained from all donors. Only male donors were used to eliminate, as a variable in the analysis, the known effects of gender on VL.[2],[19] PBMCs were isolated from venous blood by density gradient sedimentation on Ficoll-Paque Plus (GE Healthcare, Uppsala, Sweden) and cultured in RPMI 1640 (Gibco) with 20% autologous serum in 60 ml Teflon wells (Savillex Corporation) at 37°C in 5% CO_2_. Serum was obtained from the volunteers using BD Vacutainer Serum Plus Blood Collection tubes (Becton Dickson). On day 6, human MDMs were purified by adherence to tissue culture plates (Corning) that had been pre-coated with poly-*L*-lysine (0.1 mg/ml; Sigma). After 4 hours culture in RPMI 1640 with 10% heat-inactivated fetal calf serum (Sigma), 2 mM L-glutamine, 100U/ml penicillin and 100 µg/ml streptomycin (Gibco) [RP-10] at 37°C, 5% CO_2_, non-adherent lymphocytes were rinsed off. MDMs were infected with stationary phase *L. chagasi* parasites at a 10∶1 parasite:MDM ratio. Plates were immediately centrifuged at 60 *g* for 4 minutes at 4°C to synchronize the infections. After one hour, non-adherent parasites were rinsed off and cells were maintained in RP-10. PBMCs were again isolated from the same donor on day 7, and CD3^+^ cells were isolated by negative selection using either a cocktail of antibody-coated beads (anti-CD14, anti-CD19, anti-CD56; Miltenyi Biotec) or the Pan-T-Cell Isolation Kit II (Miltenyi Biotec) according to manufacturer's instructions. A small aliquot was fixed and stained for flow cytometry analysis with an anti-CD3-PE conjugated antibody (Miltenyi Biotec) to assess enrichment. The resultant enriched population should contain a mixed population of both Leishmania-naïve CD4^+^ and CD8^+^ T cells, but should be depleted of monocytes, dendritic cells, NK cells and NKT cells. Negative selection routinely resulted in a population of cells that was >90–95% CD3^+^ (data not shown). Autologous T cells were added to the infected macrophage cultures at an estimated 3∶1 T cell:MDM ratio, and the plates were again spun at 4°C and 60 *g* for 4 minutes.

### Assessment of infection

Macrophages were removed from the wells with citric saline [0.135M KCl, 0.015M Trisodium citrate); Fischer] for 5 minutes at 37°C. Infection efficiency was evaluated by manually counting ≥200 macrophages in cytospin preparations (Cytospin 4, Thermo-Shandon Fisher) stained with Diff Quik (Protocol Hema 3, Fisher Scientific). Experiments in which ≥70% macrophages were infected were used for RNA extraction. In the infection replicates used for the microarray studies, we averaged a percent infection (mean±SEM) of 79.9±7.2 for the infected macrophage only replicates and a percent infection of 76±7.7 for the infected macrophage-T-cell co-culture replicates.

### RNA isolation, cDNA preparation and hybridization

Total RNA was isolated using Trizol (Invitrogen) as specified in the manufacturer's instructions. RNA was treated with DNaseI and further cleaned using the Qiagen RNeasy mini-kit (Qiagen, Hilden, Germany). RNA for microarrays was harvested from uninfected or infected macrophages and from uninfected or infected macrophage – T-cell co-cultures at 4 hours after the initiation of co-culture (28 hours after initiation of infection). RNAs for validation experiments were harvested after 4 hours, 24 hours, 2 days and 3 days of co-culture. RNA quality was assessed using the Agilent Model 2100 Bioanalyzer (Agilent Technologies, Palo Alto, CA). cRNA was generated from five µg of total RNA by using the Affymetrix GeneChip one-cycle target labeling kit (Affymetrix, Inc., Santa Clara, CA) according to the manufacturer's recommended protocols. The resultant biotinylated cRNA was fragmented and hybridized to the GeneChip Human Genome U133 Plus 2.0 Array (Affymetrix, Inc.). The arrays were washed, stained, and scanned using the Affymetrix Model 450 Fluidics Station and Affymetrix Model 3000 scanner using the manufacturer's recommended protocols by the University of Iowa DNA Core Facility. Each sample and microarray underwent standard quality control evaluations for cRNA amplification of more than 4-fold, percentage of probe sets reliably detecting between 40 and 60 percent present call, and a 3′-5′ ratio of *GAPDH* gene less than 3.

### Microarray data analysis

Raw data analysis was performed using code written in R and software from the open-source Bioconductor Project.[20] Preliminary data quality control assessments were performed with *affyQCReport*. The raw fluorescence data were background adjusted, normalized and converted to expression-level data using *gcRMA*.[21],[22] For each donor, the log_2_ gcRMA expression data from the uninfected sample were subtracted from the log_2_ gcRMA expression data from the infected sample and then these paired data were analyzed with *RankProd* [23],[24] using the one-class model. Macrophage-only data were analyzed separately from the macrophage-T cell co-culture data. Both raw and normalized data have been deposited with ArrayExpress (http://www.ebi.ac.uk/microarray-as/aer/?#ae-main[0]) under accession number *E-MEXP-1290*. Briefly, RankProd rank orders all probe sets within each replicate by expression level and then calculates an ‘RP-Value’ for each probe set based on the amount that a particular probe set appears at the top or at the bottom of the ranked list. The RP-Value for each probe set then increases if the probe set is consistently present at the top or the bottom of the list. The software then re-sorts all the probe sets based on RP-Value, taking into account all pair-wise comparisons and adjusts for multiple hypothesis testing via permutation of the replicate labels. For each probe set, a “Percent False Positive” (PFP) value is calculated as an estimate of the false discovery rate.[23],[25] The cutoff for significance was chosen to be all genes with a PFP rate of 5% or less. These genes, adjusted by permutation for multiple hypothesis testing, had less than a 5% chance of representing a false positive signal of statistically significant differential expression. After significant genes were identified, annotation and functional clustering was performed using DAVID.[26] The raw list of AffyIDs for each condition (MDM vs. MDM-T cell co-culture; up vs. down regulated by infection) was submitted as a “Gene List” to DAVID and then the data were analyzed using the “Functional Annotation Clustering” tool using the “Highest” classification stringency setting.

Pathway analysis was performed using Gene Set Enrichment Analysis,v.2.[27] GSEA takes a list of genes and tests whether, within that queried list of genes, there is statistically significant enrichment or not of pre-defined groups of genes, or “gene sets.” Gene sets examined through GSEA include canonical metabolic and signaling pathways, groups of genes previously identified and validated to be up- or down-regulated when cells are given a particular stimulus, or genes present at a similar physical location (e.g. within a particular cytoband). This type of analysis can detect subtle changes present in the data. For example, if several key members of a particular signaling pathway are all up-regulated by 5% these changes may not be detected by traditional analyses, although these changes could biologically represent a substantial increase in the net “flux” of the signaling pathway. GSEA settings were default except for 1000 permutations, “phenotype” permutation and calculating differential expression based on the mean expression value for each phenotype.

### Real-time PCR

cDNA was generated from the cleaned up total RNA samples using the Superscript III First Strand Synthesis System kit (Invitrogen) using random hexamer primers and an RNaseH-treatment step following the manufacturer's instructions. TaqMan real-time PCR gene expression assays were purchased from Applied Biosystems, Inc. (ABI) and were performed according to the manufacturer's instructions. Data were analyzed using the Δ(ΔC_t_) method.[28]

### Cytokine assays

Supernatants from three independent infections, each from a separate individual, were assayed. The supernatants were stored at −20°C before use. Cytokine levels in the supernatants were assessed using a panel and controls samples, from Lincoplex (Millipore, Billerica, MA), according to the manufacturer's instructions. Data were generated on a BioRad Bio-Plex Assay Reader 200 (BioRad).

### Statistics

RT-PCR and cytokine data were plotted as the mean of three independent experiments. Statistical significance was assessed with GraphPad Prism, v.5 using a 1-way ANOVA and Tukey's Multiple Comparison Test or a 2-way ANOVA test.

## Results

**Figure 1:**
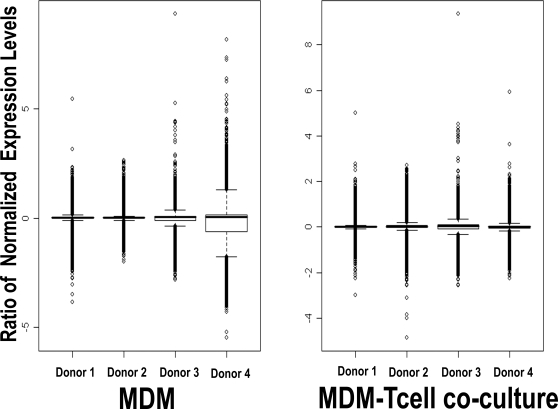
BoxPlot of Ratios [Infected:Uninfected] of gcRMA-transformed microarray expression data. The value of the log_2_(Infected expression value ÷ Uninfected expression value) for each probe set in both the MDM only condition and the MDM-T cell co-culture was calculated and plotted separately for each donor. The central box represents the interquartile range and the solid black bar represents the median probe set. Downstream analyses were performed using all four donor data sets.

To test our hypothesis that the parasite induces unique changes in gene expression in both innate and adaptive cells of the immune system encountered early in infection, we examined gene expression in four parallel conditions: (a) Uninfected MDMs, (b) Infected MDMs, (c) Uninfected MDMs co-cultured with autologous T-cells and (d) Infected MDMs co-cultured with autologous T-cells. A box plot of the log_2_ ratios of the Uninfected expression values subtracted from the Infected expression values for all probe sets plotted separately for each donor demonstrated that although there was slightly more variation present in the MDM-only ratios for Donor 4, overall there did not appear to be substantial inter-donor variation (Figure 1). Most genes did not appear to change markedly from zero as evidenced by the narrow range of most of the inter-quartile boxes. Although the analyses presented below were generated employing all 4 donors' data, the analyses were also tested leaving donor 4 out and did not differ substantially (data not shown). To generate the RNA samples, we only used infections where there was >70% infected macrophages at the 4 hour co-culture time point (MDM: 79.9±7.2% infection; MDM-T-cell: 76±7.7;see Methods). For subsequent experiments over longer time intervals, the percent of infected cells stayed roughly constant over the time intervals measured (see figure legends).

Data generated from the analysis of RNA from cultured MDMs alone were analyzed separately by RankProd from the data from MDM-T cell co-cultures. The numbers and identity of genes either up-regulated or down-regulated in infected samples compared to uninfected samples that had a particular estimated PFP were computed. In both culture conditions, statistically significant differentially-regulated genes were considered if genes met a 5% PFP cutoff. Out of 54,675 total probe sets, in the MDM only condition RankProd identified 9 probe sets with a PFP cutoff of ≤5% that were up-regulated by infection with *L. chagasi* and 72 probe sets that were down-regulated. In the MDM-T cell co-culture condition, RankProd identified 116 probe sets with a PFP cutoff of ≤ 5% that were up-regulated by infection with *L. chagasi* and 19 down-regulated probe sets. A complete list of all probe sets identified by RankProd is provided in Table S2.

After identification of the differentially expressed probe-sets using RankProd, the lists of AffyIDs for the up- and down-regulated probe-sets were submitted to the DAVID bioinformatics website to functionally cluster the various probe-sets by Gene Ontology categories and other functional annotation.[26] Tables 1 and 2 show a selected list of the identified differentially expressed genes grouped by functional annotation. In the MDM cultures (Table 1), infection led to many more down-modulated than up-regulated transcripts. In contrast to macrophage infection with bacterial pathogens,[29] Leishmania infection of MDMs did not lead to up-regulation of transcripts encoding proteins characteristic of classical inflammation. Infection with *L. chagasi* down-modulated several transcripts encoding proteins involved in cellular regulation. Down-modulated gene transcripts included those corresponding to *YAF2*, which encodes a protein that binds the transcription factor YY1, *eIF2C3*, an initiation factor belonging to the PIWI family that is essential for mammalian cell siRNA-mediated gene silencing,[30] *Cdc42*, whose protein product is involved in actin regulation, a guanine nucleotide exchange factor, and a gene involved in autophagy. There was also significant down-modulation of transcripts encoding classical inflammatory receptors (IL-1R2 and CSF2),[29] and Peroxiredoxin 6, a protein involved in redox cycling and oxidative defense.[31] Transcripts encoding proteins associated with classical macrophage activation, such as TNF-α, IL-10, MIP-1-α, IP-10, IL-6, iNOS, MHC II and CIITA,[32] were not differentially regulated.

**Table 1:** Modulation of MDM gene expression caused by *L. chagasi* infection

MDM: up-regulated transcripts (9 of 54,675 probe sets†)				
Symbol	Description	Cytoband	Fold Change	% False Positive
CXCL-5	chemokine (C-X-C motif) ligand 5	4q12-q13	2.628	0.012
**Metallothioneins**				
MT1M, F, G	metallothionein 1M, 1F, 1G	16q13	2.7–21.146	0–0.05
**Cell Metabolism**				
APOBEC-3A	apolipoprotein B mRNA editing enzyme, catalytic polypeptide-like 3A	22q13.1-q13.2	2.578	0.013
ST3GAL3	ST3 beta-galactoside alpha-2,3-sialyltransferase 3	1p34.1	5.267	0.015
AFF4	AF4/FMR2 family, member 4	5q31	1.851	0.015
IFI44L	interferon-induced protein 44-like	1p31.1	2.094	0.049
AKR1C2	aldo-keto reductase family 1, member C2	10p15-p14	1.541	0.054
**MDM: down-regulated transcripts** (selected from 72 of 54,675 probe sets†)				
**Cell Regulation**				
MLL	myeloid/lymphoid or mixed-lineage leukemia	11q23	0.373	0.016
YAF-2	YY1 associated factor 2	12q12	0.453	0.012
eIF2C3	translation initiation factor 2C	1p34.3	0.420	0.031
**Protein Metabolism**				
PHGDH	phosphoglycerate dehydrogenase	1p12	0.343	0.011
FMNL3	formin-like 3	12q13.12	0.394	0.014
DPEP2	dipeptidase 2	16q22.1	0.373	0.018
SENP6	SUMO1/sentrin specific peptidase 6	6q13-q14.3	0.455	0.040
**Immune Response**				
IL1-R2	interleukin 1 receptor, type II	2q12-q22	0.200–0.425	0–0.028
TNFSF-15	tumor necrosis factor (ligand) superfamily, member 15	9q32	0.311	0.0071
SIGLEC-10	sialic acid binding Ig-like lectin 10	19q13.3	0.374	0.014
**Kinase Activity**				
PCTK2	PCTAIRE protein kinase 2	12q23.1	0.383	0.013
PAK1	p21/Cdc42/Rac1-activated kinase 1	11q13-q14	0.396	0.015
TNFRSF-10B	tumor necrosis factor receptor superfamily, member 10b	8p22-p21	0.436	0.018
**Other**				
RAPGEF1	Rap guanine nucleotide exchange factor (GEF) 1	9q34.3	0.478	0.048
TFRC	transferrin receptor (p90, CD71)	3q29	0.357	0.012
ATG16L2	ATG16 autophagy related 16-like 2	11q13.4	0.340	0.014
PRDX6	peroxiredoxin 6	1q25.1	0.398	0.017

Genes were selected from the overall list of probe sets found to be differentially regulated by RankProd with a PFP <0.05 and functionally clustered with DAVID.

**†:** 54,675 probe sets were analyzed, and the indicated number were selected as significantly (PFP<0.05) changed between infected and noninfected macrophages.

**Table 2:** Modulation of gene expression in MDM-T cell co-cultures induced by *L. chagasi* infection

MDM-T cell co-cultures: up-regulated transcripts (selected from 116 of 54,675 probe sets†)				
Symbol	Description	Cytoband	Fold Change	% False Positive
CD-69	CD69 antigen	12p13-p12	1.724	0.010
**Metallothioneins**				
MT - 1E,1F,1G, 1H,1M,1X, 2A	metallothionein genes	16q13	3.29–37.07	0 – 0
**Inflammatory and Chemotactic Cytokines**				
IL-2	interleukin 2	4q26-q27	4.359	0
IFN-γ	interferon, gamma	12q14	2.966	0
CXCL-2,-3,-9,-10,-11	chemokine (C-X-C) ligands 2, 3, 9, 10 and 11	4q21, 4q21.2	1.62–2.29	0.049–0.008
CCL-8, -10	chemokine (C-C motif) ligands 8, -20	17q11.2	1.78–2.77	0
IL-6	interleukin 6	7p21	2.003	0.003
TNF-α	tumor necrosis factor, alpha	6p21.3	1.487	0.039
IL-1β	interleukin 1, beta	2q14	1.561	0.042
IL-1α	interleukin 1, alpha	2q14	1.611	0.042
TNFSF-10, TNFSF-11	tumor necrosis factor (ligand) superfamily, members 10 and 11	3q26, 13q14	1.69, 1.85	0.022, 0.009
**Inflammatory Mediators**				
PTGS2	prostaglandin-endoperox. synthase 2	1q25.2-q25.3	2.212	0.001
STAT1	signal transducer and activator of transcription 1	2q32.2	1.860	0.006
IFIT2, IFIT3	interferon-induced protein with tetratricopeptide repeats 2 and 3	10q23-q25	1.870; 1.786	0.008–0.010
CD-36	CD36 antigen	7q11.2	1.781	0.013
C1S	complement component subunit 1s	12p13	1.721	0.017
SOD2	superoxide dismutase 2, mitochondrial	6q25.3	1.672	0.031
CSF2	colony stimulating factor 2 (granulocyte-macrophage)	5q31.1	1.639	0.034
**Type 2 Transcripts**				
IL-4	interleukin 4	5q31.1	1.677	0.018
SOCS3	suppressor of cytokine signaling 3	17q25.3	1.653	0.025
**MDM-T cell co-culture: down-regulated transcripts** (selected from 19 of 54,675 probe sets†)				
**Symbol**	**Description**	**Cytoband**	**Fold Change**	**% False Positive**
**Immune Signaling**				
CCR-3	chemokine (C-C motif) receptor 3	3p21.3	0.427	0
IL1-R2	interleukin 1 receptor, type II	2q12-q22	0.401	0
**Cell-Cycle and Amino Acid Metabolism**				
PSAT1	phosphoserine aminotransferase 1	9q21.2	0.470	0.003
PHGDH	phosphoglycerate dehydrogenase	1p12	0.521	0.011
COL6A2	collagen, type VI, alpha 2	21q22.3	0.472	0.031
COL23A1	collagen, type XXIII, alpha 1	5q35.3	0.545	0.041
CDK-10	Cyclin-dependent kinase (CDC2-like)	16q24	0.532	0.037
GDF15	growth differentiation factor 15	19p13.1-13.2	0.542	0.039
MARS2	methionine-tRNA synthetase 2	2q33.1	0.543	0.047
**Collagen subunits**				
COL6A2	collagen, type VI, alpha 2	21q22.3	0.472	0.031
COL23A1	collagen, type XXIII, alpha 1	5q35.3	0.545	0.041

Genes were selected from the overall list of probe sets found to be differentially regulated by RankProd with a PFP <0.05 and functionally clustered with DAVID.

**†:** 54,675 probe sets were analyzed, and the indicated number were selected as significantly (PFP<0.05) changed between infected and noninfected macrophages.

The MDM-T cell co-culture condition provided a model of the initial interaction between Leishmania-infected macrophages and circulating T cells, using a mixed population of peripheral blood-derived Leishmania-naïve T cells.[33] We chose an early time point (4 hours of co-culture; 28 hours total infection time) to study the gene expression initiated by this initial contact. In contrast to our study of infected MDMs, the addition of T-cells to the co-culture increased the proportion of genes that were differentially regulated (Table 2). Of note, the mRNA encoding the early activation T-cell marker CD69 was significantly induced in the co-culture replicates, suggesting that we were able to successfully extract mRNA from both the infected macrophages as well as the added T cells. Among the many transcripts that were up-regulated by co-culture were those that encoded proteins that promote acute inflammation, including chemokines that attract neutrophils (CXCL-2, 3) and resting T cells/NK cells (CXCL-10). The latter, also called IP-10, promotes Th1-type immunity. Cytokines and interleukins expressed uniquely in the infected co-culture condition included IL-1α, IL-1β, and IL-6 which are pro-inflammatory, and IFN-γ and IL-2 which are both produced by and promote the development of a Th1-type cells. Consistently, the mRNA for *STAT1*, a key signaling molecule in the IFN-γ pathway, also increased. Transcripts encoding other chemokines (CCL-8, 20; CXCL-9, 11) were also induced in the co-culture. As such, the “flavor” of transcripts induced uniquely in co-cultures with the addition of T-cells to infected MDMs reflects an environment favorable for the development of type 1 immune cytokine responses.

Similar to the results in the MDM-only condition, transcripts for several isoforms of the MT-1 gene were highly up-regulated in co-cultures containing infected MDMs. Due to their up-regulation either with or without T-cells present, we presume these most likely reflect changes in MT gene expression occurring in the infected MDMs, although it is possible that these transcripts were also up-regulated in the co-cultured T-cells.[34]

**Figure 2:**
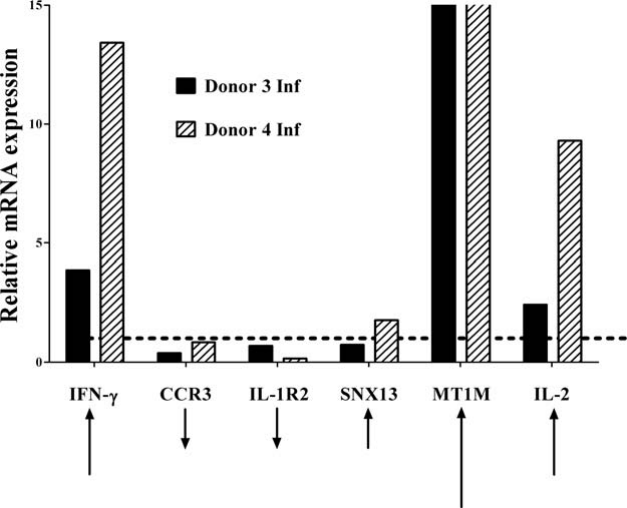
Validation of microarray data with TaqMan RT-PCR of selected differentially expressed genes. RNA was extracted from human MDM-T cell co-cultures 4 hours after the addition of T-cells to infected or uninfected macrophages (28 total hours of *L. chagasi* infection). The relative expression of genes in infected compared to uninfected levels, in two separate donors, was examined using TaqMan RT-PCR. Six genes identified to be differentially expressed in the primary microarray analysis were selected for validation. Relative expression of infected samples is displayed in reference to a line at y = 1, signifying no change. Bars below y = 1 signify genes that have decreased expression relative to uninfected samples. The black arrows at the bottom indicate the relative magnitude and direction of change anticipated from the microarray data.

In order to validate some of the above data using a more quantitative method, we performed TaqMan-based reverse transcriptase-PCR experiments on 6 differentially regulated genes. The relative mRNA expression levels were verified using the same RNA samples that had been analyzed in microarrays for two donors (Figure 2). With the exception of *SNX13* which yielded equivocal results, the direction and magnitude of calculated change comparing infected samples to uninfected samples corresponded to the change predicted by the microarray expression data for both donors. *SNX13* had been identified as an up-regulated gene by microarray analysis, but did not change appreciably above baseline by RT-PCR. In addition to the six transcripts illustrated in Figure 2, we also validated by RT-PCR the up-regulation of the metallothionein gene, *MT1M*. The relative mRNA levels of this gene were increased roughly six-fold after four hours of co-culture (data not shown).

In addition to the functional clustering using DAVID, we investigated the data using the pathway software, Gene Set Enrichment Analysis (GSEA).[27] We examined separately the MDM only and the MDM-T cell co-culture data sets. Selected results from this analysis are represented in Table S1. When corrected for multiple hypothesis testing (FDR q-value <0.02[25]), the MDM only data did not show statistically significant enrichment of any gene sets, consistent with infected MDMs exhibiting a “quiescent” phenotype. In contrast, the MDM-T cell data demonstrated enrichment of several gene sets annotated to be canonical immune cytokine signaling pathways as well as groups of genes up-regulated when different cell types are stimulated with a variety of conditions such as hypoxia, proliferation or cytokines. Of particular interest, IL-6 related gene sets appear several times on the list as KRETZSCHMAR_IL6_DIFF, BROCKE_IL6 and IL6_PATHWAY. The former two gene sets include genes that are differentially regulated when multiple myeloma cells are treated with recombinant IL-6.[35] The IL-6 Pathway and additionally the IL-12 Pathway gene sets (Biocarta) were both found to be enriched within the infected MDM-T cell co-culture data set. IL-6 mRNA itself, but not IL-12 subunits, were up-regulated in co-cultures (see Table 2). Furthermore, the GATA3 pathway (Biocarta) and TGF-β signaling pathway (Biocarta) were also enriched in the MDM-T-cell microarray data indicating that some genes not belonging to inflammatory pathways were induced. GATA3 is a transcription factor and “master regulator” of Th2 differentiation,[36] and TGF-β suppresses both Th1 and Th2 effector cell development.[37],[38] In the primary data, IL-4 mRNA was only up-regulated 1.7-fold in contrast to IFN-γ mRNA which went up almost 3-fold. In aggregate, these data suggest the infection induced primarily a type 1 immune cytokine activation phenotype, but other modulatory factors (i.e. TGF-β) may be secondarily activated.

**Figure 3:**
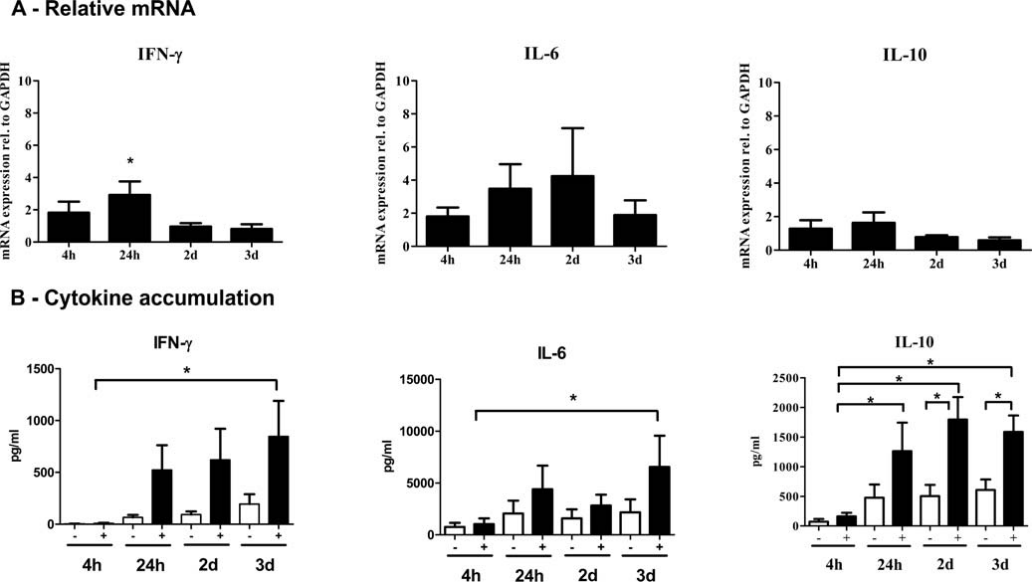
RT-PCR and cytokine assays of IFN-γ, IL-6 and IL-10 in MDM-T-cell co-cultures. MDMs were infected with *L. chagasi* for 24 hours, and autologous Leishmania-naïve T-cells were added for the indicated times. (A) Gene expression was examined comparing RNA harvested at the same time point from infected versus uninfected co-cultures. All mRNA levels were normalized to GAPDH. (B) Supernatants were collected after the indicated times of co-culture from uninfected (−) versus infected (+) MDM-T cell co-cultures. Cytokine concentrations were measured using Lincoplex (Millipore) cytokine beads. Standard curves were generated using standards provided by the company. Data for relative mRNA levels or cytokine concentrations represent the mean±SEM of three independent infection experiments from different human donors. * = p<0.05. Statistical analysis was performed using (A) 1-way ANOVA and Tukey's Multiple Comparison Test or a (B) 2-way ANOVA test, respectively.

To determine the duration of transcript and protein up-regulation, selected cytokines associated with inflammatory responses were measured. We chose IFN-γ and IL-6, whose transcripts were up-regulated according to microarray data (Table 2). We also examined IL-10, a cytokine that promotes progressive VL disease, based upon the hypothesis that important modifying factors are secondarily up-regulated in response to the initial type 1 response.[10] The relative mRNA abundance at time points between 4 h of co-culture (i.e. 28 hours of infection) and 3d of co-culture were measured using TaqMan based RT-PCR gene expression assays and normalized to *GAPDH* (Figure 3A, Δ(ΔC_t_) method). The IFN-γ mRNA peaked at 24 h at roughly three-fold above background (p<0.05) and then declined back to baseline. IL-6 mRNA showed a trend toward increased expression during infection, although the changes were not statistically significant. IL-10 mRNA did not demonstrate any significant change, consistent with our microarray findings.

Changes in the levels of IFN-γ, IL-6 and IL-10 in the supernatants of MDM-T cell co-culture wells were measured at the same time points (Figure 3B). In all cases, infected co-cultures showed significant accumulation of cytokine (p<0.05) comparing 4h of co-culture to 3d of co-culture, whereas there was no similar accumulation of cytokine in uninfected wells. IFN-γ cytokine accumulation peaked at about 4-fold above uninfected cells at 72 hours whereas IL-10 accumulated to approximately 2.5-fold above uninfected cells. The mean percent infection levels for these replicates were 83.2±2.7, 81.1±1.8, 71.2±1.3 and 63.8±7.3% at 4, 24, 48 and 72 hours of co-culture, respectively. Thus, despite the fact that the levels of IFN-γ increased out to 3d of co-culture, the percent of infected cells stayed roughly constant over the same time interval.

## Discussion

Our study was designed to test the hypothesis that a unique immune response to *L. chagasi*/*infantum* is initiated early during the initial interactions between the first immune system cells that encounter the parasite. These include macrophages and T-cells, elements of the innate and adaptive immune systems, respectively. To that end, we used microarrays to examine early gene expression patterns in purified Leishmania-naïve human T cells during their first encounter with infected human macrophages. The data suggested that macrophages exhibit a quiescent phenotype 24 hrs after infection with Leishmania, but Leishmania-naïve T cells respond to infected MDMs primarily with an inflammatory or a type 1 immune cytokine response. These *in vitro* data suggest that the initial microenvironment created at the site of Leishmania infection may be conducive to development of a type 1 adaptive immune response.

Immune responses during symptomatic VL are dominated by suppression of antigen-specific IFN-γ responses[3] and patients have high levels of the suppressive cytokines IL-10 and TGF-β in their serum with a negative Montenegro reactions.[8]–[10],[13],[19] Whether this immunosuppression initiates early in the process of macrophage:T cell interactions is not fully known.

Several studies have previously profiled transcriptional responses of phagocytic cells to Leishmania infection. Buates and Matlashewski[14] showed that in *L. donovani* infected BALB/c macrophages, ∼40% of the examined genes in a modified array are down-regulated 4 hours after infection. Rodriguez et al.[39] reported that murine macrophages exhibited a novel non-classical, non-alternative activation profile at early time points after *L. chagasi* infection. More recently, Chaussabel et al.[40] used microarrays to compare gene expression in human macrophages or dendritic cells infected for 16 hours with a variety of parasitic and bacterial pathogens including *L. major* and *L. donovani*. These authors showed that Leishmania infection invokes the expression of a novel set of genes that is Leishmania species-specific. Notably, *L. major*-infected macrophages down-regulated IFN-γ induced genes yet overall induce a stronger inflammatory profile than does *L. donovani*. Both Leishmania species induce *IL6* gene expression.

Prior studies of PBMCs incubated with species of *Leishmania* causing cutaneous leishmaniasis, called *in vitro* priming systems, demonstrated the prominent production of type 1 cytokines (IL-12 and IFN-γ) but lower levels of type 2 cytokines such as IL-5.[41] In contrast, infection of PBMCs with *L. donovani*, which causes visceral leishmaniasis, inhibits the of production of pro-inflammatory cytokines such as IL-1 or TNF-α[42] and leads to interruption of IFN-γ signaling pathways.[43] During the current study, we pursued a global analysis of gene expression shortly after human peripheral blood derived macrophages first encounter Leishmania-naïve T-cells. Based on previously published work from our laboratory, promastigotes convert to amastigotes by 24–48 hours after infection of human macrophages.[44] Furthermore, during infection of murine macrophages with wild-type *L. chagasi,* fusion of developing phagosomes with lysosomes is delayed for 24–48 hours.[45] At the 28 hour post infection time point examined in this assay, it is therefore reasonable to assume that most if not all parasites will have converted to amastigotes and reside within phagolysosomes.

Examination of genes expressed in the infected-macrophages-only condition revealed more genes were down-regulated than were induced. Macrophage activation patterns can be divided into classical, alternative, and Type II activation. Alternatively activated macrophages up-regulate IL-1RA, mannose receptor (MRC1), scavenger receptor (CD36), the low-affinity IgE receptor (CD23) and exhibit high arginase activity. Type II macrophages up-regulate sphingosine kinase 1 (SPHK1), LIGHT, TNF-SF14, FIZZ1 and IL-10, have high NO**^.^** production but remain arginase low.[32],[39],[46] Of the above characteristic macrophage activation transcripts, none were significantly up- or down-regulated after 28 hours of *L. chagasi* infection. It is possible that some of the above defining macrophage activation markers would have been up- or down-regulated if samples had been taken earlier after the initiation of infection. Nonetheless, the time point chosen for this study captured macrophages harboring converted intracellular amastigotes[44] and the MDM alone condition allowed us to compare background gene expression by infected macrophages with the macrophage-T cell co-culture condition.

To our surprise, and in contrast with the quiescent phenotype of the infected-macrophages, four hours after the addition of Leishmania-naïve T cells to infected macrophages, multiple genes characteristic of a type 1 immune cytokine response were up-regulated. Highlights of up-regulated transcripts included IFN-γ, STAT-1, IL-1α, IL-1β, TNF-α and IL-6. Further bioinformatics analysis using GSEA confirmed the fact that genes and pathways initiated by pro-inflammatory cytokines and chemokines were up-regulated. Additionally, using GSEA we also observed enrichment of the TGF-β pathway; a cytokine that is suppressive of both Th1-type and Th2-type immune responses.[37],[38] Although we cannot discern exactly which cell type (MDM or T cell) contributed most highly to the above transcripts, comparison with the MDM alone conditions suggests that at least some of the cytokines such as IFN-γ may have been derived from T-cells. It should be emphasized that other cell types such as dendritic cells and NK cells should have been largely excluded from the co-culture through the positive selection on the purification column.

Direct measurements of mRNA and protein levels in the co-cultured infected macrophages and T cells showed that the steady state abundance of both the mRNA and protein of IFN-γ and IL-6 accumulated significantly by 3d in the co-cultures (Figure 3). Although *IL10* mRNA did not appreciably increase at 3d of infection, we observed a significant increase in levels of IL-10 in culture supernatants after 3 days of co-cultivation (Figure 3). The infection-induced accumulation of IL-6 and IFN-γ would be predicted by their respective mRNA abundance, whereas the accumulation of IL-10 was unexpected. This finding could reflect that we missed a transient peak of *IL-10* mRNA during MDM infection. Nonetheless since the IL-10 pathway was not enriched using GSEA, it is reasonable to hypothesize that the accumulation of IL-10 could be a secondary, modifying response to the increase in IFN-γ and IL-6 rather than a primary response to Leishmania infected macrophages. The same scenario could be hypothesized for TGF-β.

The present data suggest that the initial interactions of *L. chagasi*-infected macrophages with the adaptive immune system results primarily in up-regulation of type 1 immune cytokine responses. There was little evidence for type 2 activation, as IL-4 was only up-regulated slightly less than two-fold in the primary microarray co-culture data and characteristic type 2 chemokine receptors such as CCR3[47] were, in fact, down-regulated. It remains to be determined at what point *L. chagasi* parasites begin to tip the balance of immunity away from a curative type 1, IFN-γ response to cause symptomatic disease.

## Supporting Information

Supplementary Table S1 Gene Sets enriched during *L. chagasi* infection of MDM-T-cell co-cultures according to the Genes Set Enrichment Analysis (GSEA).
(0.12 MB DOC)

Supplementary Table S2 Complete list of all probe sets identified by RankProd as differentially regulated upon infection by *L. chagasi* for both the MDM-only and MDM-T co-culture conditions.
(0.46 MB DOC)
