# Supplementary material for: Macrophage and T-Cell Gene Expression in a Model of Early Infection with the Protozoan Leishmania chagasi
Source: PLoS Negl Trop Dis. 2008 Jun 25;2(6):e252. doi: 10.1371/journal.pntd.0000252 (PMC2427198; doi:10.1371/journal.pntd.0000252)
Supplement: Supplementary Table S1 — Gene Sets enriched during L. chagasi infection of MDM-T-cell co-cultures according to the Genes Set Enrichment Analysis (GSEA). (0.12 MB DOC) [file pntd.0000252.s001.doc]

**Supplementary Table S1:** Gene Sets enriched during *L. chagasi* infection of MDM-T-cell co-cultures according to the Genes Set Enrichment Analysis (GSEA).*,^¶^

| **Gene Set Name^†^** | **Description** | **Set Size^‡^** | **NES^‡^** | **NOM p-val^§^** | **FDR q-val^¶^** |
| --- | --- | --- | --- | --- | --- |
| HALMOS_CEBP_UP | Up-regulated genes following C/EBP induction | 50 | 2.342 | 0 | 0.015 |
| BRCA2_BRCA1_DN | Genes down-regulated in BRCA2 vs. BRCA1 tumors | 42 | 2.334 | 0 | 0.015 |
| FSH_OVARY_MCV152_UP | FSH-treated ovarian epithelial cells. | 61 | 2.281 | 0 | 0.015 |
| HDACI_COLON_CLUSTER6 | Genes upregulated in colon carcinoma cells after treatment with HDACs. | 43 | 2.276 | 0 | 0.015 |
| P53HYPOXIAPATHWAY | p53 - Biocarta | 19 | 2.268 | 0 | 0.015 |
| BRENTANI_SIGNALING | Cancer related genes involved in the cell signaling | 178 | 2.259 | 0 | 0.015 |
| KRETZSCHMAR_IL6_DIFF | IL-6 treated INA-6 cells. | 145 | 2.259 | 0 | 0.015 |
| BROCKE_IL6 | IL-6 treated INA-6 cells. | 145 | 2.259 | 0 | 0.017 |
| GATA3PATHWAY | Gata3 - Biocarta | 15 | 2.244 | 0 | 0.015 |
| BRENTANI_TRANSCRIPTION_FACTORS | Cancer related genes that are also transcription factors | 64 | 2.228 | 0 | 0.015 |
| SIG_PIP3_SIGNALING_IN_B_LYMPHOCYTES | Genes related to PIP_3_ signaling in B cells (curated) | 35 | 2.226 | 0 | 0.015 |
| IL6PATHWAY | IL-6 - Biocarta | 21 | 2.225 | 0 | 0.015 |
| MENSE_HYPOXIA_UP | Hypoxia-induced genes in Astrocytes and HeLa cells | 107 | 2.225 | 0 | 0.015 |
| BIOPEPTIDESPATHWAY | G-protein receptors - Biocarta | 38 | 2.213 | 0 | 0.015 |
| CREBPATHWAY | CREB - Biocarta | 27 | 2.208 | 0 | 0.015 |
| IL1_CORNEA_UP | IL-1 treatment of corneal fibroblasts | 62 | 2.207 | 0 | 0.015 |
| CMV_HCMV_TIMECOURSE_8HRS_UP | hCMV infected fibroblasts | 20 | 2.201 | 0 | 0.015 |
| ST_GA12_PATHWAY | Signaling Transduction KE | 21 | 2.197 | 0 | 0.015 |
| LINDSTEDT_DEND_8H_VS_48H_UP | Early stimulated DC genes | 64 | 2.189 | 0 | 0.015 |
| BRG1_ALAB_UP | AV-mediated over-expression of mutant BRG1 in ALAB cells | 40 | 2.188 | 0 | 0.015 |
| FETAL_LIVER_ENRICHED_TRANSCRIPTION_FACTORS | Transcription factors enriched in fetal liver - Jean-Pierre Bourquin | 75 | 2.168 | 0 | 0.015 |
| OKUMURA_MC_LPS | LPS treated mast cells | 185 | 2.165 | 0 | 0.015 |
| PPARAPATHWAY | PPARα - Biocarta | 54 | 2.164 | 0 | 0.015 |
| LAIRPATHWAY | Acute inflammation - Biocarta | 15 | 2.153 | 0 | 0.017 |
| IFN_BETA_UP | IFN-β treated HT1080 cells | 65 | 2.153 | 0 | 0.017 |
| BYSTROM_IL5_DN | IL-5 treated mouse bone marrow | 57 | 2.150 | 0 | 0.017 |
| TOB1PATHWAY | TOB1 – Biocarta | 17 | 2.150 | 0 | 0.017 |
| ADDYA_K562_HEMIN_TREATMENT | Hemin treated K562 cells | 70 | 2.130 | 0 | 0.016 |
| HADDAD_HPCLYMPHO_ENRICHED | Umbilical cord blood progenitor cells vs. common lymphoid progenitors | 309 | 2.128 | 0 | 0.016 |
| LEE_TCELLS7_UP | CD4^+^ cells vs. other T cells | 15 | 2.126 | 0 | 0.016 |
| SIG_BCR_SIGNALING_PATHWAY | BCR pathway - SignalingAlliance | 45 | 2.124 | 0 | 0.016 |
| RUTELLA_HEPATGFSNDCS_UP | HGF treated monocytes | 158 | 2.121 | 0 | 0.016 |
| TCELL_ANERGIC_UP | Anergic mouse T CD4^+^ (A.E7) cells vs. non-anergic stimulated cells | 81 | 2.120 | 0 | 0.016 |
| CALCINEURIN_NF_AT_SIGNALING | Calcineurin - GEArray | 93 | 2.115 | 0 | 0.016 |
| KNUDSEN_PMNS_UP | Migrating skin lesion PMNs | 74 | 2.112 | 0 | 0.016 |
| OXSTRESS_BREASTCA_UP | Oxidatively stressed breast cancer cells | 29 | 2.111 | 0 | 0.016 |
| HADDAD_HSC_CD10_UP | Umbilical cord blood progenitor cells vs. common lymphoid progenitors | 296 | 2.108 | 0 | 0.016 |
| HADDAD_CD45CD7_PLUS_VS_MINUS_DN | Umbilical cord blood progenitor cells vs. common lymphoid progenitors | 84 | 2.104 | 0 | 0.016 |
| HADDAD_HSC_CD7_DN | Umbilical cord blood progenitor cells vs. common lymphoid progenitors | 84 | 2.104 | 0 | 0.016 |
| TPA_SENS_EARLY_DN | TPA treated HL-60 cells | 284 | 2.097 | 0 | 0.016 |
| IL1RPATHWAY | IL-1R – Biocarta | 31 | 2.094 | 0 | 0.017 |
| TPOPATHWAY | TPO – Biocarta | 23 | 2.094 | 0 | 0.017 |
| GSK3PATHWAY | Gsk3-β - Biocarta | 26 | 2.087 | 0 | 0.017 |
| CHIARETTI_T_ALL | T cell ALL cells | 255 | 2.085 | 0 | 0.017 |
| PENG_GLUCOSE_DN | Glucose starvation of lymphocytes | 134 | 2.080 | 0 | 0.017 |
| IFN_ANY_UP | IFN treatment of HT1080 cells | 81 | 2.074 | 0 | 0.017 |
| IL2RBPATHWAY | IL-2 – Biocarta | 34 | 2.071 | 0 | 0.017 |
| VIPPATHWAY | VIP - Biocarta | 27 | 2.069 | 0 | 0.017 |
| ST_ADRENERGIC | Signaling Transduction KE | 33 | 2.067 | 0 | 0.017 |
| PASSERINI_PROLIFERATION | Cell adhesion related genes in endothelial cells | 64 | 2.058 | 0 | 0.017 |
| HSC_HSC_FETAL | Mouse hematopoietic stem cells | 237 | 2.057 | 0 | 0.017 |
| HSC_LTHSC_FETAL | Long-term functional liver hematopoietic stem cells | 268 | 2.048 | 0 | 0.016 |
| HSC_LTHSC_SHARED | Long-term functional liver hematopoietic stem cells | 268 | 2.048 | 0 | 0.017 |
| CYTOKINEPATHWAY | Cytokines – Biocarta | 20 | 2.042 | 0 | 0.016 |
| DER_IFNB_UP | IFN-β treated HT1080 cells | 93 | 2.042 | 0 | 0.016 |
| KIM_TH_CELLS_DN | Germinal center CD4^+^ cells versus other CD4^+^ cells | 15 | 2.038 | 0 | 0.016 |
| NFATPATHWAY | NFAT - Biocarta | 52 | 2.034 | 0 | 0.016 |
| PASSERINI_SIGNAL | Signaling related genes in endothelial cells | 338 | 2.028 | 0 | 0.017 |
| HIVNEFPATHWAY | HIV-Nef - Biocarta | 55 | 2.018 | 0 | 0.017 |
| TGF_BETA_SIGNALING_PATHWAY | TGF-β - Broad Institute | 49 | 2.017 | 0 | 0.017 |
| BRENTANI_IMMUNE_FUNCTION | Cancer related genes involved in immune function | 50 | 2.012 | 0 | 0.017 |
| SA_PTEN_PATHWAY | PTEN - SigmaAldrich | 17 | 2.011 | 0 | 0.017 |
| REOVIRUS_HEK293_UP | Reovirus infected Hek293 cells | 236 | 2.010 | 0 | 0.017 |
| IL12PATHWAY | IL-12 - Biocarta | 20 | 2.003 | 0 | 0.018 |
| INSULINPATHWAY | Insulin - Biocarta | 21 | 2.001 | 0 | 0.018 |
| IGF1PATHWAY | IGF-1 – Biocarta | 20 | 2.001 | 0.11 | 0.018 |
| PASSERINI_APOPTOSIS | Apoptosis related genes in endothelial cells | 43 | 2.000 | 0 | 0.018 |

* Analysis of MDMs cultured with or without *L. chagasi* infection did not yield significant enrichment of pathways.

^†^ See Results section for details of datasets investigated. Complete lists of all genes within each gene set are available at the MSigDB section of the GSEA website (<http://www.broad.mit.edu/gsea/msigdb/genesets.jsp>). Website is free but users must register first.

^‡^ NES – normalized enrichment score; Set Size - number of genes in the gene set after filtering out those genes not in the expression dataset. Please see the GSEA User Guide or Subramanian et al.^[27]^ for further definitions and algorithm details.

^§^ NOM p-value – nominal (uncorrected) p-value. Please see the GSEA User Guide or Subramanian et al.^[27]^ for further definitions and algorithm details.

^¶^ Gene sets with an FDR q-value < 0.02 corrected for multiple testing^[25]^and an NES *>* 2^[27]^ are shown.
