# Supplementary material for: Macrophage and T-Cell Gene Expression in a Model of Early Infection with the Protozoan Leishmania chagasi
Source: PLoS Negl Trop Dis. 2008 Jun 25;2(6):e252. doi: 10.1371/journal.pntd.0000252 (PMC2427198; doi:10.1371/journal.pntd.0000252)
Supplement: Supplementary Table S2 — Complete list of all probe sets identified by RankProd as differentially regulated upon infection by L. chagasi for both the MDM-only and MDM-T co-culture conditions. (0.46 MB DOC) [file pntd.0000252.s002.doc]

**Supplementary Table S2: Complete RankProd Results**

| **MDM ONLY** | | | | | | | | | |
| --- | --- | --- | --- | --- | --- | --- | --- | --- | --- |
| **Up-Regulated** | | | |  |  | |  |  | |
| **Probe** | **Symbol** | **Description** | | **Cytoband** | **Entrez Gene #** | | **Fold Chg** | **% False Pos.** | |
| 217546_at | MT1M | metallothionein 1M | | 16q13 | 4499 | | 21.145 | 0.000 | |
| 214974_x_at | CXCL5 | chemokine (C-X-C motif) ligand 5 | | 4q12-q13 | [6374](http://www.ncbi.nlm.nih.gov/sites/entrez?cmd=retrieve&db=gene&uid=6374) | | 2.628 | 0.012 | |
| 204745_x_at | MT1G | metallothionein 1G | | 16q13 | [4495](http://www.ncbi.nlm.nih.gov/sites/entrez?cmd=retrieve&db=gene&uid=4495) | | 3.282 | 0.013 | |
| 210873_x_at | APOBEC3A | apolipoprotein B mRNA editing enzyme, catalytic polypeptide-like 3A | | 22q13.1-q13.2 | [200315](http://www.ncbi.nlm.nih.gov/sites/entrez?cmd=retrieve&db=gene&uid=200315) | | 2.578 | 0.013 | |
| 1555181_a_at | ST3GAL3 | ST3 beta-galactoside alpha-2,3-sialyltransferase 3 | | 1p34.1 | [6487](http://www.ncbi.nlm.nih.gov/sites/entrez?cmd=retrieve&db=gene&uid=6487) | | 5.267 | 0.015 | |
| 239439_at | AFF4 | AF4/FMR2 family, member 4 | | 5q31 | [27125](http://www.ncbi.nlm.nih.gov/sites/entrez?cmd=retrieve&db=gene&uid=27125) | | 1.851 | 0.015 | |
| 217165_x_at | MT1F | metallothionein 1F (functional) | | 16q13 | [4494](http://www.ncbi.nlm.nih.gov/sites/entrez?cmd=retrieve&db=gene&uid=4494) | | 2.716 | 0.048 | |
| 204439_at | IFI44L | interferon-induced protein 44-like | | 1p31.1 | [10964](http://www.ncbi.nlm.nih.gov/sites/entrez?cmd=retrieve&db=gene&uid=10964) | | 2.094 | 0.049 | |
| 211653_x_at | AKR1C2 | aldo-keto reductase family 1, member C2 (dihydrodiol dehydrogenase 2, bile acid binding protein, 3-alpha hydroxysteroid dehydrogenase, type III) | | 10p15-p14 | [1646](http://www.ncbi.nlm.nih.gov/sites/entrez?cmd=retrieve&db=gene&uid=1646) | | 1.541 | 0.054 | |
|  | | | | | | | | | |
| **MDM ONLY** | | | | | | | | | |
| **Down-Regulated** | | | |  |  | |  |  | |
| **Probe** | **Symbol** | **Description** | | **Cytoband** | **Entrez Gene #** | | **Fold Chg** | **% False Pos.** | |
| 228416_at | ORC4L | origin recognition complex, subunit 4-like (yeast) | | 2q22-q23 | [5000](http://www.ncbi.nlm.nih.gov/sites/entrez?cmd=retrieve&db=gene&uid=5000) | | 0.354 | 0.000 | |
| 242945_at | FAM20A | family with sequence similarity 20, member A | | 17q24.2 | [54757](http://www.ncbi.nlm.nih.gov/sites/entrez?cmd=retrieve&db=gene&uid=54757) | | 0.307 | 0.000 | |
| 229168_at | COL23A1 | collagen, type XXIII, alpha 1 | | 5q35.3 | [91522](http://www.ncbi.nlm.nih.gov/sites/entrez?cmd=retrieve&db=gene&uid=91522) | | 0.289 | 0.000 | |
| 219341_at | CLN8 | ceroid-lipofuscinosis, neuronal 8 (epilepsy, progressive with mental retardation) | | 8p23 | [2055](http://www.ncbi.nlm.nih.gov/sites/entrez?cmd=retrieve&db=gene&uid=2055) | | 0.286 | 0.000 | |
| 226663_at | ANKRD10 | ankyrin repeat domain 10 | | 13q34 | [55608](http://www.ncbi.nlm.nih.gov/sites/entrez?cmd=retrieve&db=gene&uid=55608) | | 0.278 | 0.000 | |
| 211372_s_at | IL1R2 | interleukin 1 receptor, type II | | 2q12-q22 | [7850](http://www.ncbi.nlm.nih.gov/sites/entrez?cmd=retrieve&db=gene&uid=7850) | | 0.200 | 0.000 | |
| 221085_at | TNFSF15 | tumor necrosis factor (ligand) superfamily, member 15 | | 9q32 | [9966](http://www.ncbi.nlm.nih.gov/sites/entrez?cmd=retrieve&db=gene&uid=9966) | | 0.311 | 0.007 | |
| 201730_s_at | TPR | translocated promoter region (to activated MET oncogene) | | 1q25 | [7175](http://www.ncbi.nlm.nih.gov/sites/entrez?cmd=retrieve&db=gene&uid=7175) | | 0.311 | 0.010 | |
| 1557418_at | ACSL4 | acyl-CoA synthetase long-chain family member 4 | | Xq22.3-q23 | [2182](http://www.ncbi.nlm.nih.gov/sites/entrez?cmd=retrieve&db=gene&uid=2182) | | 0.340 | 0.011 | |
| 201397_at | PHGDH | phosphoglycerate dehydrogenase | | 1p12 | [26227](http://www.ncbi.nlm.nih.gov/sites/entrez?cmd=retrieve&db=gene&uid=26227) | | 0.343 | 0.011 | |
| 243561_at | YAF2 | YY1 associated factor 2 | | 12q12 | [10138](http://www.ncbi.nlm.nih.gov/sites/entrez?cmd=retrieve&db=gene&uid=10138) | | 0.453 | 0.012 | |
| 239022_at | TFRC | transferrin receptor (p90, CD71) | | 3q29 | [7037](http://www.ncbi.nlm.nih.gov/sites/entrez?cmd=retrieve&db=gene&uid=7037) | | 0.357 | 0.012 | |
| 215123_at | LOC348162 |  | | 16p11.2 | [348162](http://www.ncbi.nlm.nih.gov/sites/entrez?cmd=retrieve&db=gene&uid=348162) | | 0.397 | 0.013 | |
| 235652_at | SCML1 | sex comb on midleg-like 1 (Drosophila) | | Xp22.2-p22.1 | [6322](http://www.ncbi.nlm.nih.gov/sites/entrez?cmd=retrieve&db=gene&uid=6322) | | 0.400 | 0.013 | |
| 237459_at | PCTK2 | PCTAIRE protein kinase 2 | | 12q23.1 | [5128](http://www.ncbi.nlm.nih.gov/sites/entrez?cmd=retrieve&db=gene&uid=5128) | | 0.383 | 0.013 | |
| 223482_at | TMPIT |  | | 7q11.23 | [83862](../83862) | | 0.352 | 0.013 | |
| 1552807_a_at | SIGLEC10 | sialic acid binding Ig-like lectin 10 | | 19q13.3 | [89790](../89790) | | 0.374 | 0.014 | |
| 229389_at | ATG16L2 | ATG16 autophagy related 16-like 2 (S. cerevisiae) | | 11q13.4 | [89849](http://www.ncbi.nlm.nih.gov/sites/entrez?cmd=retrieve&db=gene&uid=89849) | | 0.340 | 0.014 | |
| 238823_at | FMNL3 | formin-like 3 | | 12q13.12 | [91010](http://www.ncbi.nlm.nih.gov/sites/entrez?cmd=retrieve&db=gene&uid=91010) | | 0.394 | 0.014 | |
| 211948_x_at | BAT2D1 | BAT2 domain containing 1 | | 1q23.3 | [23215](http://www.ncbi.nlm.nih.gov/sites/entrez?cmd=retrieve&db=gene&uid=23215) | | 0.347 | 0.014 | |
| 230100_x_at | PAK1 | p21/Cdc42/Rac1-activated kinase 1 (STE20 homolog, yeast) | | 11q13-q14 | [5058](http://www.ncbi.nlm.nih.gov/sites/entrez?cmd=retrieve&db=gene&uid=5058) | | 0.396 | 0.015 | |
| 207038_at | SLC16A6 | solute carrier family 16 (monocarboxylic acid transporters), member 6 | | 17q24.2 | [9120](http://www.ncbi.nlm.nih.gov/sites/entrez?cmd=retrieve&db=gene&uid=9120) | | 0.379 | 0.015 | |
| 1562062_at | LOC200030 |  | | 1q21.1 | [200030](http://www.ncbi.nlm.nih.gov/sites/entrez?cmd=retrieve&db=gene&uid=200030) | | 0.499 | 0.016 | |
| 1565651_at |  |  | |  |  | | 0.426 | 0.016 | |
| 1563321_s_at | MLL | myeloid/lymphoid or mixed-lineage leukemia (trithorax homolog, Drosophila) | | 11q23 | [4297](http://www.ncbi.nlm.nih.gov/sites/entrez?cmd=retrieve&db=gene&uid=4297) | | 0.373 | 0.016 | |
| 230703_at | C14orf32 | chromosome 14 open reading frame 32 | | 14q22.2-q22.3 | [93487](http://www.ncbi.nlm.nih.gov/sites/entrez?cmd=retrieve&db=gene&uid=93487) | | 0.367 | 0.016 | |
| 228694_at |  |  | |  |  | | 0.391 | 0.017 | |
| 242751_at | PRDX6 | peroxiredoxin 6 | | 1q25.1 | [9588](http://www.ncbi.nlm.nih.gov/sites/entrez?cmd=retrieve&db=gene&uid=9588) | | 0.398 | 0.017 | |
| 230028_at | PPP1R13B | protein phosphatase 1, regulatory (inhibitor) subunit 13B | | 14q32.33 | [23368](http://www.ncbi.nlm.nih.gov/sites/entrez?cmd=retrieve&db=gene&uid=23368) | | 0.357 | 0.017 | |
| 227077_at | ZNF286 | zinc finger protein 286 | | 17p11.2 | [57335](http://www.ncbi.nlm.nih.gov/sites/entrez?cmd=retrieve&db=gene&uid=57335) | | 0.386 | 0.018 | |
| 229826_at | LOC440957 |  | | 3p21.1 | [440957](http://www.ncbi.nlm.nih.gov/sites/entrez?cmd=retrieve&db=gene&uid=440957) | | 0.422 | 0.018 | |
| 209294_x_at | TNFRSF10B | tumor necrosis factor receptor superfamily, member 10b | | 8p22-p21 | [8795](http://www.ncbi.nlm.nih.gov/sites/entrez?cmd=retrieve&db=gene&uid=8795) | | 0.436 | 0.018 | |
| 219452_at | DPEP2 | dipeptidase 2 | | 16q22.1 | [64174](http://www.ncbi.nlm.nih.gov/sites/entrez?cmd=retrieve&db=gene&uid=64174) | | 0.373 | 0.018 | |
| 240013_at |  |  | |  |  | | 0.405 | 0.018 | |
| 222413_s_at | MLL3 | myeloid/lymphoid or mixed-lineage leukemia 3 | | 7q34-q36 | [58508](http://www.ncbi.nlm.nih.gov/sites/entrez?cmd=retrieve&db=gene&uid=58508) | | 0.395 | 0.019 | |
| 1556423_at | VASH1 | vasohibin 1 | | 14q24.3 | [22846](http://www.ncbi.nlm.nih.gov/sites/entrez?cmd=retrieve&db=gene&uid=22846) | | 0.419 | 0.019 | |
| 205403_at | IL1R2 | interleukin 1 receptor, type II | | 2q12-q22 | [7850](http://www.ncbi.nlm.nih.gov/sites/entrez?cmd=retrieve&db=gene&uid=7850) | | 0.425 | 0.028 | |
| 65585_at | FLJ10661 |  | | 11q13.4 | [55199](http://www.ncbi.nlm.nih.gov/sites/entrez?cmd=retrieve&db=gene&uid=55199) | | 0.411 | 0.031 | |
| 214807_at |  |  | |  |  | | 0.423 | 0.031 | |
| 219426_at | EIF2C3 | eukaryotic translation initiation factor 2C, 3 | | 1p34.3 | [192669](http://www.ncbi.nlm.nih.gov/sites/entrez?cmd=retrieve&db=gene&uid=192669) | | 0.420 | 0.031 | |
| 207057_at | SLC16A7 | solute carrier family 16 (monocarboxylic acid transporters), member 7 | | 12q13 | [9194](http://www.ncbi.nlm.nih.gov/sites/entrez?cmd=retrieve&db=gene&uid=9194) | | 0.474 | 0.031 | |
| 1557996_at |  |  | |  |  | | 0.503 | 0.032 | |
| 214241_at | NDUFB8 | NADH dehydrogenase (ubiquinone) 1 beta subcomplex, 8, 19kDa | | 10q23.2-q23.33 | [4714](http://www.ncbi.nlm.nih.gov/sites/entrez?cmd=retrieve&db=gene&uid=4714) | | 0.457 | 0.032 | |
| 224563_at | WASF2 | WAS protein family, member 2 | | 1p36.11-p34.3 | [10163](http://www.ncbi.nlm.nih.gov/sites/entrez?cmd=retrieve&db=gene&uid=10163) | | 0.455 | 0.032 | |
| 244341_at | MAK3 | Mak3 homolog (S. cerevisiae) | | 3q13.2 | [80218](http://www.ncbi.nlm.nih.gov/sites/entrez?cmd=retrieve&db=gene&uid=80218) | | 0.433 | 0.032 | |
| 225509_at | SAP30L |  | | 5q33.2 | [79685](http://www.ncbi.nlm.nih.gov/sites/entrez?cmd=retrieve&db=gene&uid=79685) | | 0.423 | 0.032 | |
| 1558515_at |  |  | |  |  | | 0.466 | 0.035 | |
| 215029_at | C1orf108 | chromosome 1 open reading frame 108 | | 1p34.3 | [79647](http://www.ncbi.nlm.nih.gov/sites/entrez?cmd=retrieve&db=gene&uid=79647) | | 0.453 | 0.037 | |
| 1570078_a_at | DOCK5 | dedicator of cytokinesis 5 | | 8p21.2 | [80005](http://www.ncbi.nlm.nih.gov/sites/entrez?cmd=retrieve&db=gene&uid=80005) | | 0.462 | 0.037 | |
| 243768_at | SENP6 | SUMO1/sentrin specific peptidase 6 | | 6q13-q14.3 | [26054](http://www.ncbi.nlm.nih.gov/sites/entrez?cmd=retrieve&db=gene&uid=26054) | | 0.455 | 0.040 | |
| 213089_at | LOC340089 |  | | 5q13.2 | [340089](http://www.ncbi.nlm.nih.gov/sites/entrez?cmd=retrieve&db=gene&uid=340089) | | 0.455 | 0.040 | |
| 236524_at | TM2D1 | TM2 domain containing 1 | | 1p31.3 | [83941](http://www.ncbi.nlm.nih.gov/sites/entrez?cmd=retrieve&db=gene&uid=83941) | | 0.484 | 0.040 | |
| 1554599_x_at | LOC440426 |  | | 17q12 | [440426](http://www.ncbi.nlm.nih.gov/sites/entrez?cmd=retrieve&db=gene&uid=440426) | | 0.413 | 0.040 | |
| 229202_at | PCNXL2 | pecanex-like 2 (Drosophila) | | 1q42.2 | [80003](../80003) | | 0.458 | 0.041 | |
| 204083_s_at | TPM2 | tropomyosin 2 (beta) | | 9p13.2-p13.1 | [7169](http://www.ncbi.nlm.nih.gov/sites/entrez?cmd=retrieve&db=gene&uid=7169) | | 0.462 | 0.041 | |
| 220694_at | DDEF1IT1 | DDEF1 intronic transcript 1 | | 8q24.21 | [29065](http://www.ncbi.nlm.nih.gov/sites/entrez?cmd=retrieve&db=gene&uid=29065) | | 0.419 | 0.041 | |
| 207735_at | RNF125 | ring finger protein 125 | | 18q12.1 | [54941](http://www.ncbi.nlm.nih.gov/sites/entrez?cmd=retrieve&db=gene&uid=54941) | | 0.403 | 0.042 | |
| 210340_s_at | CSF2RA | colony stimulating factor 2 receptor, alpha, low-affinity (granulocyte-macrophage) | | Xp22.32 and Yp11.3 | [1438](http://www.ncbi.nlm.nih.gov/sites/entrez?cmd=retrieve&db=gene&uid=1438) | | 0.409 | 0.043 | |
| 242691_at |  |  | |  |  | | 0.517 | 0.044 | |
| 222787_s_at | FLJ11273 |  | | 7p21.3 | 54664 | | 0.420 | 0.048 | |
| 235028_at |  |  | |  |  | | 0.471 | 0.048 | |
| 221577_x_at | GDF15 | growth differentiation factor 15 | | 19p13.1-13.2 | 9518 | | 0.425 | 0.048 | |
| 220892_s_at | PSAT1 | phosphoserine aminotransferase 1 | | 9q21.2 | 29968 | | 0.460 | 0.048 | |
| 207186_s_at | FALZ | fetal Alzheimer antigen | | 17q24.3 | 2186 | | 0.394 | 0.048 | |
| 244408_at | RAPGEF1 | Rap guanine nucleotide exchange factor (GEF) 1 | | 9q34.3 | 2889 | | 0.478 | 0.048 | |
| 219221_at | ZBTB38 | zinc finger and BTB domain containing 38 | | 3q23 | 253461 | | 0.434 | 0.049 | |
| 1554154_at | GDAP2 | ganglioside induced differentiation associated protein 2 | | 1p12 | 54834 | | 0.434 | 0.049 | |
| 235409_at | MGA | MAX gene associated | | 15q14 | 23269 | | 0.413 | 0.049 | |
| 217197_x_at | CG018 |  | | 13q12-q13 | 90634 | | 0.446 | 0.049 | |
| 220261_s_at | ZDHHC4 | zinc finger, DHHC-type containing 4 | | 7p22.1 | 55146 | | 0.433 | 0.049 | |
| 227463_at | ACE | angiotensin I converting enzyme (peptidyl-dipeptidase A) 1 | | 17q23 | 1636 | | 0.495 | 0.050 | |
| 202379_s_at | NKTR | natural killer-tumor recognition sequence | | 3p23-p21 | 4820 | | 0.452 | 0.050 | |
|  | | | | | | | | | |
| **MDM-T cell co-culture** | | | | | | | | | |
| **Up-Regulated** | | |  | | |  |  | |  |
| **Probe** | **Symbol** | **Description** | **Cytoband** | | | **Entrez Gene #** | **Fold Chg** | | **% False Pos.** |
| 217546_at | MT1M | metallothionein 1M | 16q13 | | | 4499 | 37.068 | | 0.000 |
| 212859_x_at | MT1E | metallothionein 1E (functional) | 16q13 | | | 4493 | 4.739 | | 0.000 |
| 207849_at | IL2 | interleukin 2 | 4q26-q27 | | | 3558 | 4.359 | | 0.000 |
| 204745_x_at | MT1G | metallothionein 1G | 16q13 | | | 4495 | 3.949 | | 0.000 |
| 213629_x_at | MT1F | metallothionein 1F (functional) | 16q13 | | | 4494 | 3.426 | | 0.000 |
| 217165_x_at | MT1F | metallothionein 1F (functional) | 16q13 | | | 4494 | 3.404 | | 0.000 |
| 216336_x_at | MT1M | metallothionein 1M | 16q13 | | | 4499 | 3.358 | | 0.000 |
| 206461_x_at | MT1H | metallothionein 1H | 16q13 | | | 4496 | 3.356 | | 0.000 |
| 208581_x_at | MT1X | metallothionein 1X | 16q13 | | | 4501 | 3.291 | | 0.000 |
| 204326_x_at | MT1X | metallothionein 1X | 16q13 | | | 4501 | 3.286 | | 0.000 |
| 210354_at | IFNG | interferon, gamma | 12q14 | | | 3458 | 2.966 | | 0.000 |
| 214038_at | CCL8 | chemokine (C-C motif) ligand 8 | 17q11.2 | | | 6355 | 2.766 | | 0.000 |
| 205476_at | CCL20 | chemokine (C-C motif) ligand 20 | 2q33-q37 | | | 6364 | 2.653 | | 0.000 |
| 211122_s_at | CXCL11 | chemokine (C-X-C motif) ligand 11 | 4q21.2 | | | 6373 | 2.633 | | 0.000 |
| 211456_x_at | LOC440737 |  | 1q43 | | | 440737 | 3.130 | | 0.001 |
| 204748_at | PTGS2 | prostaglandin-endoperoxide synthase 2 (prostaglandin G/H synthase and cyclooxygenase) | 1q25.2-q25.3 | | | 5743 | 2.212 | | 0.001 |
| 210163_at | CXCL11 | chemokine (C-X-C motif) ligand 11 | 4q21.2 | | | 6373 | 2.104 | | 0.001 |
| 239979_at | EPSTI1 | epithelial stromal interaction 1 (breast) | 13q13.3 | | | 94240 | 2.087 | | 0.002 |
| 204614_at | SERPINB2 | serpin peptidase inhibitor, clade B (ovalbumin), member 2 | 18q21.3 | | | 5055 | 2.370 | | 0.002 |
| 238439_at | ANKRD22 | ankyrin repeat domain 22 | 10q23.31 | | | 118932 | 2.095 | | 0.002 |
| 202933_s_at | YES1 | v-yes-1 Yamaguchi sarcoma viral oncogene homolog 1 | 18p11.31-p11.21 | | | 7525 | 2.108 | | 0.002 |
| 231578_at | GBP1 | guanylate binding protein 1, interferon-inducible, 67kDa | 1p22.2 | | | 2633 | 2.037 | | 0.003 |
| 205207_at | IL6 | interleukin 6 (interferon, beta 2) | 7p21 | | | 3569 | 2.003 | | 0.003 |
| 210524_x_at |  |  |  | | |  | 2.897 | | 0.003 |
| 210029_at | INDO | indoleamine-pyrrole 2,3 dioxygenase | 8p12-p11 | | | 3620 | 2.020 | | 0.003 |
| 232397_at |  |  |  | | |  | 1.924 | | 0.003 |
| 212185_x_at | MT2A | metallothionein 2A | 16q13 | | | 4502 | 2.461 | | 0.003 |
| 238581_at | GBP5 | guanylate binding protein 5 | 1p22.2 | | | 115362 | 1.889 | | 0.003 |
| 210873_x_at | APOBEC3A | apolipoprotein B mRNA editing enzyme, catalytic polypeptide-like 3A | 22q13.1-q13.2 | | | 200315 | 1.856 | | 0.006 |
| 1569095_at |  |  |  | | |  | 1.872 | | 0.006 |
| 209498_at | CEACAM1 | carcinoembryonic antigen-related cell adhesion molecule 1 (biliary glycoprotein) | 19q13.2 | | | 634 | 2.083 | | 0.006 |
| 209969_s_at | STAT1 | signal transducer and activator of transcription 1, 91kDa | 2q32.2 | | | 6772 | 1.860 | | 0.006 |
| 1553575_at |  |  |  | | |  | 2.086 | | 0.006 |
| 236244_at | HNRPU | heterogeneous nuclear ribonucleoprotein U (scaffold attachment factor A) | 1q44 | | | 3192 | 1.988 | | 0.008 |
| 209774_x_at | CXCL2 | chemokine (C-X-C motif) ligand 2 | 4q21 | | | 2920 | 2.294 | | 0.008 |
| 205220_at | GPR109B | G protein-coupled receptor 109B | 12q24.31 | | | 8843 | 1.786 | | 0.008 |
| 226757_at | IFIT2 | interferon-induced protein with tetratricopeptide repeats 2 | 10q23-q25 | | | 3433 | 1.870 | | 0.008 |
| 224802_at | NDFIP2 | Nedd4 family interacting protein 2 | 13q31.1 | | | 54602 | 1.868 | | 0.009 |
| 242625_at | RSAD2 | radical S-adenosyl methionine domain containing 2 | 2p25.2 | | | 91543 | 1.677 | | 0.009 |
| 213418_at | HSPA6 | heat shock 70kDa protein 6 (HSP70B') | 1q23 | | | 3310 | 1.958 | | 0.009 |
| 205890_s_at | UBD | ubiquitin D | 6p21.3 | | | 10537 | 1.866 | | 0.009 |
| 210643_at | TNFSF11 | tumor necrosis factor (ligand) superfamily, member 11 | 13q14 | | | 8600 | 1.855 | | 0.009 |
| 229450_at | IFIT3 | interferon-induced protein with tetratricopeptide repeats 3 | 10q24 | | | 3437 | 1.786 | | 0.010 |
| 204439_at | IFI44L | interferon-induced protein 44-like | 1p31.1 | | | 10964 | 1.760 | | 0.010 |
| 219836_at | ZBED2 | zinc finger, BED-type containing 2 | 3q13.13 | | | 79413 | 1.737 | | 0.010 |
| 209795_at | CD69 | CD69 antigen (p60, early T-cell activation antigen) | 12p13-p12 | | | 969 | 1.724 | | 0.010 |
| 228439_at | MGC20410 |  | 11q13.1 | | | 116071 | 1.552 | | 0.010 |
| 204533_at | CXCL10 | chemokine (C-X-C motif) ligand 10 | 4q21 | | | 3627 | 1.776 | | 0.010 |
| 228193_s_at | RGC32 |  | 13q14.11 | | | 28984 | 1.862 | | 0.010 |
| 213550_s_at | NDUFA2 | NADH dehydrogenase (ubiquinone) 1 alpha subcomplex, 2, 8kDa | 5q31 | | | 4695 | 2.001 | | 0.010 |
| 1557718_at | PPP2R5C | protein phosphatase 2, regulatory subunit B (B56), gamma isoform | 14q32 | | | 5527 | 1.831 | | 0.011 |
| AFFX-HUMRGE/M10098_5_at |  |  |  | | |  | 1.416 | | 0.011 |
| 204747_at | IFIT3 | interferon-induced protein with tetratricopeptide repeats 3 | 10q24 | | | 3437 | 1.652 | | 0.011 |
| 1569003_at | TMEM49 | transmembrane protein 49 | 17q23.2 | | | 81671 | 1.968 | | 0.011 |
| 1552908_at | C1orf150 | chromosome 1 open reading frame 150 | 1q44 | | | 148823 | 1.785 | | 0.011 |
| 228673_s_at | EML4 | echinoderm microtubule associated protein like 4 | 2p22-p21 | | | 27436 | 1.995 | | 0.012 |
| 228766_at | CD36 | CD36 antigen (collagen type I receptor, thrombospondin receptor) | 7q11.2 | | | 948 | 1.781 | | 0.013 |
| 242727_at | ARL8 | ADP-ribosylation factor-like 8 | 10p12.31 | | | 221079 | 1.703 | | 0.013 |
| 224375_at |  |  |  | | |  | 1.783 | | 0.015 |
| 227055_at | MGC17301 |  | 12q13.2 | | | 196410 | 1.718 | | 0.015 |
| 213954_at | KIAA0888 |  | 5q13.3 | | | 26049 | 1.909 | | 0.016 |
| 208747_s_at | C1S | complement component 1, s subcomponent | 12p13 | | | 716 | 1.721 | | 0.017 |
| 207539_s_at | IL4 | interleukin 4 | 5q31.1 | | | 3565 | 1.677 | | 0.018 |
| 217502_at | IFIT2 | interferon-induced protein with tetratricopeptide repeats 2 | 10q23-q25 | | | 3433 | 1.674 | | 0.018 |
| 238725_at |  |  |  | | |  | 1.692 | | 0.020 |
| 232744_x_at | LOC360030 |  | 12p13.31 | | | 360030 | 1.747 | | 0.022 |
| 210837_s_at | PDE4D | phosphodiesterase 4D, cAMP-specific (phosphodiesterase E3 dunce homolog, Drosophila) | 5q12 | | | 5144 | 1.765 | | 0.022 |
| 239196_at | ANKRD22 | ankyrin repeat domain 22 | 10q23.31 | | | 118932 | 1.715 | | 0.022 |
| 202687_s_at | TNFSF10 | tumor necrosis factor (ligand) superfamily, member 10 | 3q26 | | | 8743 | 1.694 | | 0.022 |
| 206157_at | PTX3 | pentraxin-related gene, rapidly induced by IL-1 beta | 3q25 | | | 5806 | 1.737 | | 0.023 |
| 1559777_at |  |  |  | | |  | 1.678 | | 0.024 |
| 227697_at | SOCS3 | suppressor of cytokine signaling 3 | 17q25.3 | | | 9021 | 1.653 | | 0.025 |
| 214329_x_at | TNFSF10 | tumor necrosis factor (ligand) superfamily, member 10 | 3q26 | | | 8743 | 1.659 | | 0.025 |
| 202193_at | LIMK2 | LIM domain kinase 2 | 22q12.2 | | | 3985 | 1.667 | | 0.025 |
| 231972_at |  |  |  | | |  | 1.833 | | 0.026 |
| 213350_at | RPS11 | ribosomal protein S11 | 19q13.3 | | | 6205 | 1.773 | | 0.026 |
| 213716_s_at | SECTM1 | secreted and transmembrane 1 | 17q25 | | | 6398 | 1.635 | | 0.026 |
| 1557236_at |  |  |  | | |  | 1.633 | | 0.028 |
| 209699_x_at | AKR1C2 | aldo-keto reductase family 1, member C2 (dihydrodiol dehydrogenase 2, bile acid binding protein, 3-alpha hydroxysteroid dehydrogenase, type III) | 10p15-p14 | | | 1646 | 1.703 | | 0.028 |
| 237347_at | C6orf148 | chromosome 6 open reading frame 148 | 6q13 | | | 80759 | 1.705 | | 0.029 |
| AFFX-HUMISGF3A/M97935_MA_at | STAT1 | signal transducer and activator of transcription 1, 91kDa | 2q32.2 | | | 6772 | 1.592 | | 0.030 |
| 232375_at | STAT1 | signal transducer and activator of transcription 1, 91kDa | 2q32.2 | | | 6772 | 1.618 | | 0.031 |
| 1566342_at | SOD2 | superoxide dismutase 2, mitochondrial | 6q25.3 | | | 6648 | 1.672 | | 0.031 |
| AFFX-HUMISGF3A/M97935_5_at | STAT1 | signal transducer and activator of transcription 1, 91kDa | 2q32.2 | | | 6772 | 1.620 | | 0.032 |
| 213797_at | RSAD2 | radical S-adenosyl methionine domain containing 2 | 2p25.2 | | | 91543 | 1.468 | | 0.032 |
| AFFX-HUMISGF3A/M97935_MB_at | STAT1 | signal transducer and activator of transcription 1, 91kDa | 2q32.2 | | | 6772 | 1.594 | | 0.032 |
| 230333_at | SAT | spermidine/spermine N1-acetyltransferase | Xp22.1 | | | 6303 | 1.659 | | 0.032 |
| 206133_at | BIRC4BP |  | 17p13.1 | | | 54739 | 1.658 | | 0.032 |
| 206914_at | CRTAM |  | 11q22-q23 | | | 56253 | 1.634 | | 0.032 |
| 215671_at | PDE4B | phosphodiesterase 4B, cAMP-specific (phosphodiesterase E4 dunce homolog, Drosophila) | 1p31 | | | 5142 | 1.728 | | 0.033 |
| 222802_at |  |  |  | | |  | 1.715 | | 0.034 |
| 202581_at | HSPA1B | heat shock 70kDa protein 1B | 6p21.3 | | | 3304 | 1.593 | | 0.034 |
| 203455_s_at | SAT | spermidine/spermine N1-acetyltransferase | Xp22.1 | | | 6303 | 1.572 | | 0.034 |
| 222881_at | HPSE | heparanase | 4q21.3 | | | 10855 | 1.729 | | 0.034 |
| 236924_at | GLMN | glomulin, FKBP associated protein | 1p22.1 | | | 11146 | 1.700 | | 0.034 |
| 232197_x_at | ARSB | arylsulfatase B | 5p11-q13|5q11-q13 | | | 411 | 1.674 | | 0.034 |
| 230170_at | OSM | oncostatin M | 22q12.2 | | | 5008 | 1.624 | | 0.034 |
| 237009_at | CD69 | CD69 antigen (p60, early T-cell activation antigen) | 12p13-p12 | | | 969 | 1.751 | | 0.034 |
| 210229_s_at | CSF2 | colony stimulating factor 2 (granulocyte-macrophage) | 5q31.1 | | | 1437 | 1.639 | | 0.034 |
| 216841_s_at | SOD2 | superoxide dismutase 2, mitochondrial | 6q25.3 | | | 6648 | 1.602 | | 0.034 |
| 1552626_a_at | DKFZP566N034 |  | 2q21.3 | | | 81615 | 1.612 | | 0.035 |
| 214041_x_at | RPL37A | ribosomal protein L37a | 2q35 | | | 6168 | 1.625 | | 0.036 |
| 203915_at | CXCL9 | chemokine (C-X-C motif) ligand 9 | 4q21 | | | 4283 | 1.617 | | 0.039 |
| 207113_s_at | TNF | tumor necrosis factor (TNF superfamily, member 2) | 6p21.3 | | | 7124 | 1.487 | | 0.039 |
| 229541_at | FLJ21616 |  | 8p21.1 | | | 79618 | 1.730 | | 0.041 |
| 217441_at | USP33 | ubiquitin specific peptidase 33 | 1p31.1 | | | 23032 | 1.601 | | 0.041 |
| 39402_at | IL1B | interleukin 1, beta | 2q14 | | | 3553 | 1.561 | | 0.042 |
| 210118_s_at | IL1A | interleukin 1, alpha | 2q14 | | | 3552 | 1.611 | | 0.042 |
| 213317_at | CLIC5 | chloride intracellular channel 5 | 6p12.1-21.1 | | | 53405 | 1.611 | | 0.042 |
| 214149_s_at | ATP6V0E | ATPase, H+ transporting, lysosomal 9kDa, V0 subunit e | 5q35.1 | | | 8992 | 1.688 | | 0.043 |
| 243287_s_at | OSTM1 | osteopetrosis associated transmembrane protein 1 | 6q21 | | | 28962 | 1.798 | | 0.043 |
| 240046_at |  |  |  | | |  | 1.724 | | 0.047 |
| 1556202_at | LOC440608 |  |  | | | 440608 | 1.695 | | 0.047 |
| 207850_at | CXCL3 | chemokine (C-X-C motif) ligand 3 | 4q21 | | | 2921 | 1.785 | | 0.050 |
| 215064_at | SC5DL | sterol-C5-desaturase (ERG3 delta-5-desaturase homolog, fungal)-like | 11q23.3 | | | 6309 | 1.540 | | 0.050 |
| 240287_at | LOC341720 |  | 13q22.3 | | | 341720 | 1.532 | | 0.050 |
|  |  |  |  | | |  |  | |  |
| **MDM-T cell co-culture** | | | | | | | | | |
| **Down-Regulated** | | |  | | |  |  | |  |
| **Probe** | **Symbol** | **Description** | **Cytoband** | | | **Entrez Gene #** | **Fold Chg** | | **% False Pos.** |
| 208304_at | CCR3 | chemokine (C-C motif) receptor 3 | 3p21.3 | | | 1232 | 0.427 | | 0.000 |
| 211372_s_at | IL1R2 | interleukin 1 receptor, type II | 2q12-q22 | | | 7850 | 0.401 | | 0.000 |
| 216361_s_at | MYST3 | MYST histone acetyltransferase (monocytic leukemia) 3 | 8p11 | | | 7994 | 0.399 | | 0.000 |
| 220892_s_at | PSAT1 | phosphoserine aminotransferase 1 | 9q21.2 | | | 29968 | 0.470 | | 0.003 |
| 223062_s_at | PSAT1 | phosphoserine aminotransferase 1 | 9q21.2 | | | 29968 | 0.497 | | 0.010 |
| 201397_at | PHGDH | phosphoglycerate dehydrogenase | 1p12 | | | 26227 | 0.521 | | 0.011 |
| 219629_at | C22orf8 | chromosome 22 open reading frame 8 | 22q13 | | | 55007 | 0.478 | | 0.012 |
| 206392_s_at | RARRES1 | retinoic acid receptor responder (tazarotene induced) 1 | 3q25.32 | | | 5918 | 0.496 | | 0.012 |
| 38487_at | STAB1 | stabilin 1 | 3p21.1 | | | 23166 | 0.494 | | 0.014 |
| 218051_s_at | FLJ12442 |  | 3p21.1 | | | 64943 | 0.531 | | 0.028 |
| 209156_s_at | COL6A2 | collagen, type VI, alpha 2 | 21q22.3 | | | 1292 | 0.472 | | 0.031 |
| 219592_at | MCPH1 | microcephaly, primary autosomal recessive 1 | 8p23.1 | | | 79648 | 0.539 | | 0.032 |
| 224673_at | LENG8 | leukocyte receptor cluster (LRC) member 8 | 19q13.42 | | | 114823 | 0.559 | | 0.036 |
| 210622_x_at | CDK10 | cyclin-dependent kinase (CDC2-like) 10 | 16q24 | | | 8558 | 0.532 | | 0.037 |
| 221577_x_at | GDF15 | growth differentiation factor 15 | 19p13.1-13.2 | | | 9518 | 0.542 | | 0.039 |
| 229168_at | COL23A1 | collagen, type XXIII, alpha 1 | 5q35.3 | | | 91522 | 0.545 | | 0.041 |
| 237542_at | PRKCQ | protein kinase C, theta | 10p15 | | | 5588 | 0.521 | | 0.043 |
| 221022_s_at | PMFBP1 | polyamine modulated factor 1 binding protein 1 | 16q22.2 | | | 83449 | 0.566 | | 0.046 |
| 243529_at | MARS2 | methionine-tRNA synthetase 2 (mitochondrial) | 2q33.1 | | | 92935 | 0.543 | | 0.047 |
